# Data of ureagenesis from ammonia, glutamine and alanine, and mitochondrial aquaporin-8 expression in thioacetamide-treated hepatocytes

**DOI:** 10.1016/j.dib.2020.105632

**Published:** 2020-04-30

**Authors:** Alejo M. Capiglioni, María de Luján Alvarez, Raúl A. Marinelli

**Affiliations:** Instituto de Fisiología Experimental, Consejo Nacional de Investigaciones Científicas y Técnicas (CONICET), Facultad de Ciencias Bioquímicas y Farmacéuticas, Universidad Nacional de Rosario, Rosario, Argentina. Suipacha 570, 2000 Rosario, Santa Fe, Argentina

**Keywords:** Ureagenesis, Ammonia, Glutamine, Alanine, Thioacetamide, Mitochondrial aquaporin-8, TAA, thioacetamide, mtAQP8, mitochondrial aquaporin-8

## Abstract

We present data about the synthesis of urea from different substrates, i.e., free ammonia, glutamine and alanine in primary cultured rat hepatocytes treated or untreated with the model hepatotoxic agent thioacetamide (TAA). We also provide data about the expression of mitochondrial aquaporin-8 (mtAQP8), a hepatocyte channel protein which facilitates ammonia diffusion into mitochondria to supply the urea cycle. Ammonia-derived ureagenesis was significantly inhibited by about 30% while that from the both amino acids resulted unaffected in TAA-treated hepatocytes. Protein expression of mtAQP8 was decreased by about 80% after TAA treatment. These data can be useful for the understanding of the mechanisms of drug-induced hepatic dysfunction.

**Specifications Table**

**Table:** 

Subject	Hepatology
Specific subject area	Drug-induced hepatic dysfunction
Type of data	Figure
How data were acquired	For quantitative colorimetric urea determination was used a urea assay kit (Abnova). Absorbance was measured at 405 nm at 37°C in an automatic microplate reader (Beckman Coulter DTX 880 Multimode Detector) equipped with a thermally controlled compartment. For immunoblotting data, blots were incubated with primary antibodies against aquaporin-8 (AQP8) and prohibitin and then with horseradish peroxidase-conjugated corresponding secondary antibodies (see below for details). Bands were detected by enhanced chemiluminescence detection system and autoradiographs were obtained by exposing polyvinyl difluoride membranes to radiographic film. Densitometric analysis was performed using Image J Software.
Data format	Analyzed Raw (Supplementary data)
Parameters for data collection	Freshly isolated rat hepatocytes were cultured and exposed to thioacetamide (TAA) for 24 h. After that time, the cells were washed and exposed to ammonia, glutamine or alanine for another 24 h. Urea concentration was assessed in culture media. AQP8 protein expression was assessed in hepatocyte mitochondrial (mt) fractions.
Description of data collection	By measuring urea content in culture media with commercial kit and urea assay standard curves. By assessing protein expression of mtAQP8 using immunoblotting and densitometry.
Data source location	Rosario, Santa Fe, Argentina
Data accessibility	With the article

## Value of the data

• The data are useful because they provide information about the effect of a model hepatotoxic agent on ureagenesis from different substrates and mtAQP8 protein expression.
• The data can be relevant in studies directed towards understanding the mechanisms by which xenobiotics affect hepatic ammonia detoxification and ureagenesis.
• Researchers who study mechanisms of drug-induced hepatic dysfunction may benefit from the data.

## 1 Data Description

**Fig. 1:**
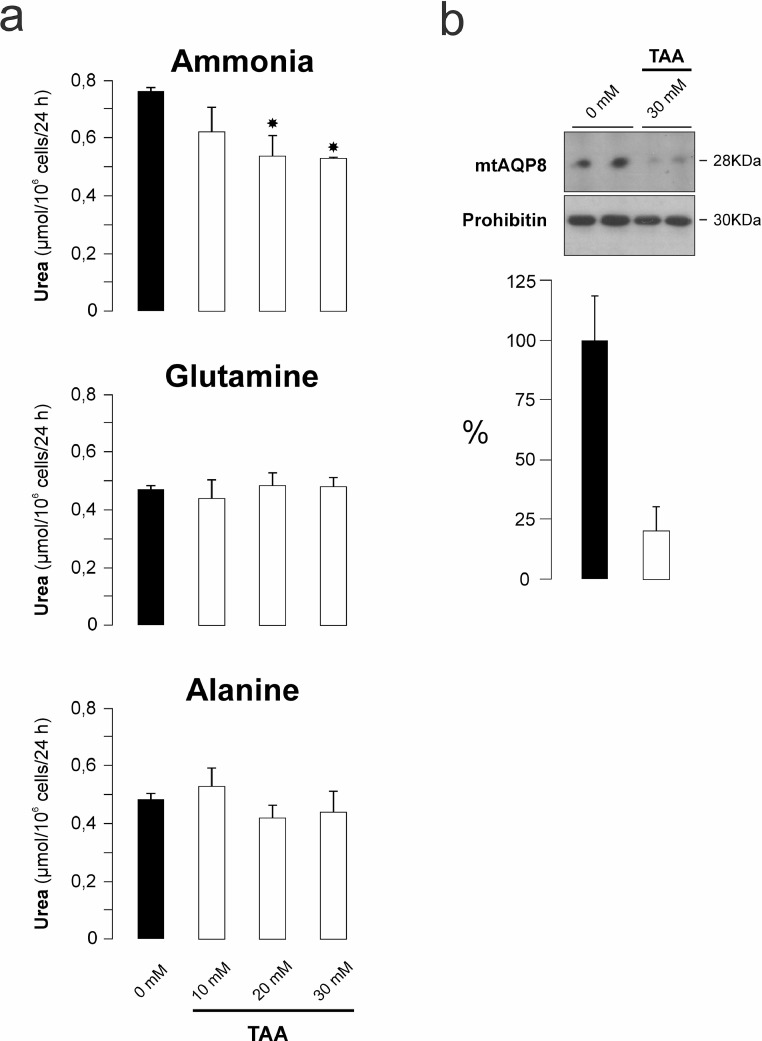
Effect of thioacetamide (TAA) on ureagenesis from free ammonia, glutamine and alanine, and mitochondrial aquaporin-8 (mtAQP8) protein expression in primary cultured rat hepatocytes. Hepatocytes were incubated with TAA (0-30 mM) for 24 h and then for another 24 h with substrates for ureagenesis as indicated in Materials and Methods. *A*) Urea production rate was determined in the culture media. Data are means ± SEM of 4-6 independent experiments in each experimental group.*P < 0.05 from 0 mM TAA. *B*) Immunoblotting for mtAQP8 protein in mitochondrial fractions isolated from hepatocytes at the end of the experiments, i.e. after the 48 h of culture. The densitometric analysis of mtAQP8 protein expression is related to Prohibitin, a mitochondrial marker. Each lane was loaded with 25 µg of protein. Data are means ± SEM of two independent experiments.

Here we show data of ureagenesis from free ammonia, glutamine and alanine in primary cultured rat hepatocytes treated with the model hepatotoxic agent, thioacetamide (TAA) (0-30 mM). Ammonia-derived urea showed a significant dose-dependent decrease of about 30% at 30 mM TAA (Fig. 1A). In contrast, ureagenesis from aminoacids, glutamine and alanine, was not significantly affected (Fig. 1A). In Fig. 1B, we show data of the protein expression of mitochondrial aquaporin-8 (mtAQP8), a channel protein which facilitates ammonia diffusion into mitochondria to supply urea cycle [1,2]. The mtAQP8 protein expression showed a reduction of about 80% in TAA-treated hepatocytes.

## 2 Experimental Design, Materials, and Methods

### 2.1 Materials and reagents

Dulbecco's Modified Eagle Medium, Pen-Strep antibiotic mixture, L-glutamine, 0.25 % Trypsin-EDTA were all from Invitrogen Corp., CA, USA. Foetal calf serum was purchased from Internegocios S.A. laboratories, Argentina. Collagenase type IV, the protease inhibitor Phenyl-methylsulfonyl fluoride, L-alanine, ammonium chloride, and TAA were from Sigma, MO, USA. Leupeptin was from Chemicon Millipore (Darmstadt, Germany). Sucrose was from MP Biomedicals, OH, USA. Polyscreen polyvinyl difluoride transfer membranes for immunoblotting were from Perkin Elmer Life and Analytical Sciences, MA, USA. Radiographic ECL hyperfilms were from Amersham GE Healthcare.

### 2.2 Isolation, culture, and treatment of rat hepatocytes

Animals received human care, according to the *Guide for the Care and Use of Laboratory Animals* (NIH). Protocols complied with the local guidelines for the use of experimental animals. Hepatocytes were isolated from normal livers of male Wistar rats by collagenase perfusion and mechanical disruption [1]. Cell viability (assessed by Trypan blue exclusion) was greater than 90%. One million hepatocytes were plated onto collagen-coated plates in Dulbecco's Modified Eagle Medium (high glucose, with 1 mM sodium pyruvate, and without L-glutamine), supplemented with 10 % heat-inactivated foetal calf serum, penicillin (100 units/ml), and streptomycin (100 μg/ml). Cells were incubated at 37°C in a humidified atmosphere with 5% CO_2_ for 3 h, allowing cell attachment to plates. Medium was changed, and hepatocytes were exposed to TAA (0-30 mM) for 24 h. After that time, cells were washed and exposed to 1 mM ammonium chloride, 4 mM L-glutamine, or 4 mM alanine for another 24 h. Urea synthesis was assessed as described below.

### 2.3 Hepatocyte urea production

It is of note that culture media contain pyruvate as source of aspartate since the urea molecule has two nitrogens, one coming from ammonia and the other from aspartate [1,2]. At the end of the experiments, the culture medium was aspirated and centrifuged at 500 *g* for 5 min to obtain a cell-free supernatant for urea determination by the urea assay kit (Abnova, Taipei, Taiwan) according to the manufacturer's instructions [3]. Hepatocytes were washed and sonicated in 0.3 M sucrose and subjected to low-speed centrifugation to obtain post-nuclear supernatants which were then centrifuged at 6,000 *g* for 10 min at 4 °C, yielding the mitochondrial fraction. Total proteins were determined according to Lowry et al [4].

### 2.4 Immunoblotting

Mitochondrial fractions were used for mtAQP8 immunoblotting as described [1]. Blots were incubated with mouse anti-AQP8 (SC 14-Z) affinity purified antibody (0.2 μg/ml, Santa Cruz Biotechnology Inc., Santa Cruz, CA). For loading control, we used rabbit antibody against prohibitin (0.1 μg/ml, Abcam, Cambridge, UK). The blots were then washed and incubated with horseradish peroxidase-conjugated corresponding secondary antibodies (Thermo), and bands were detected by enhanced chemiluminescence detection system (ECL; Amersham Pharmacia Biotech). Autoradiographs were obtained by exposing polyvinyl difluoride membranes to radiographic films. Densitometric analysis was performed using Image J Software. Under the working conditions used, there was a linear range of response of the films.

### 2.5 Statistical analysis

Data are expressed as means ± SEM. Significance was determined using Mann–Whitney test. *P* < 0.05 was considered as statistically significant.

## Appendix Supplementary materials

Image, application 1

Image, application 2
