# Supplementary figures and images for: Data of ureagenesis from ammonia, glutamine and alanine, and mitochondrial aquaporin-8 expression in thioacetamide-treated hepatocytes
Source: Data Brief. 2020 Apr 30;30:105632. doi: 10.1016/j.dib.2020.105632 (PMC7210421; doi:10.1016/j.dib.2020.105632)

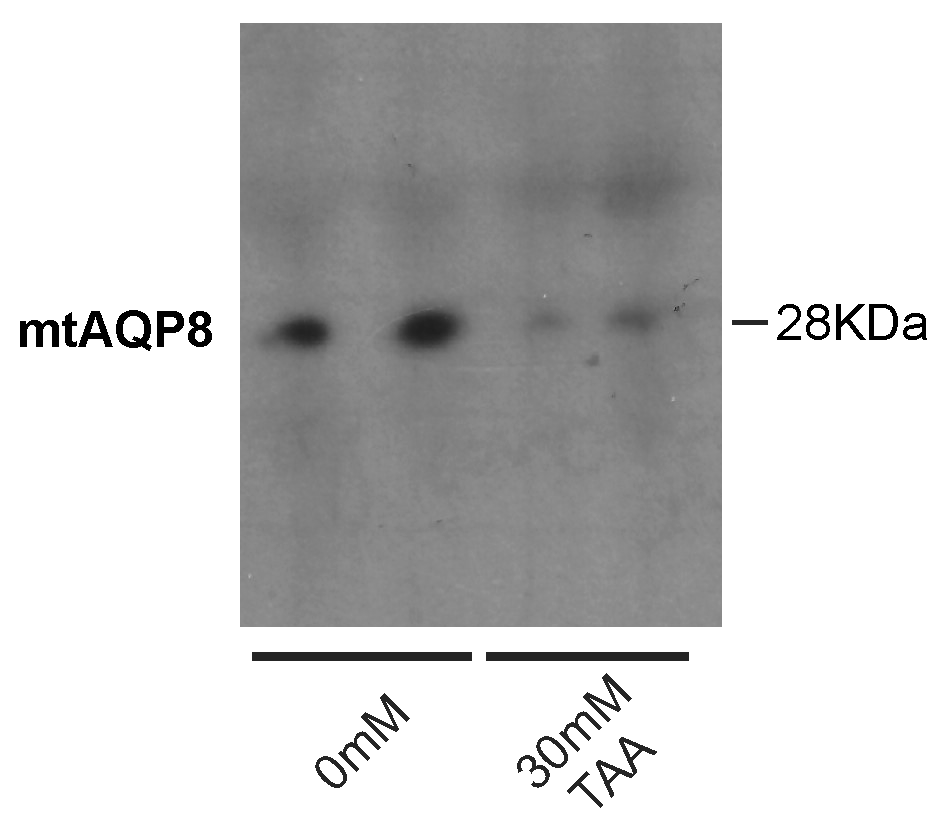

Supplement: Supplementary file 2 [file mmc2.zip › Raw data Fig.1B immunoblotting AQP8.tif]

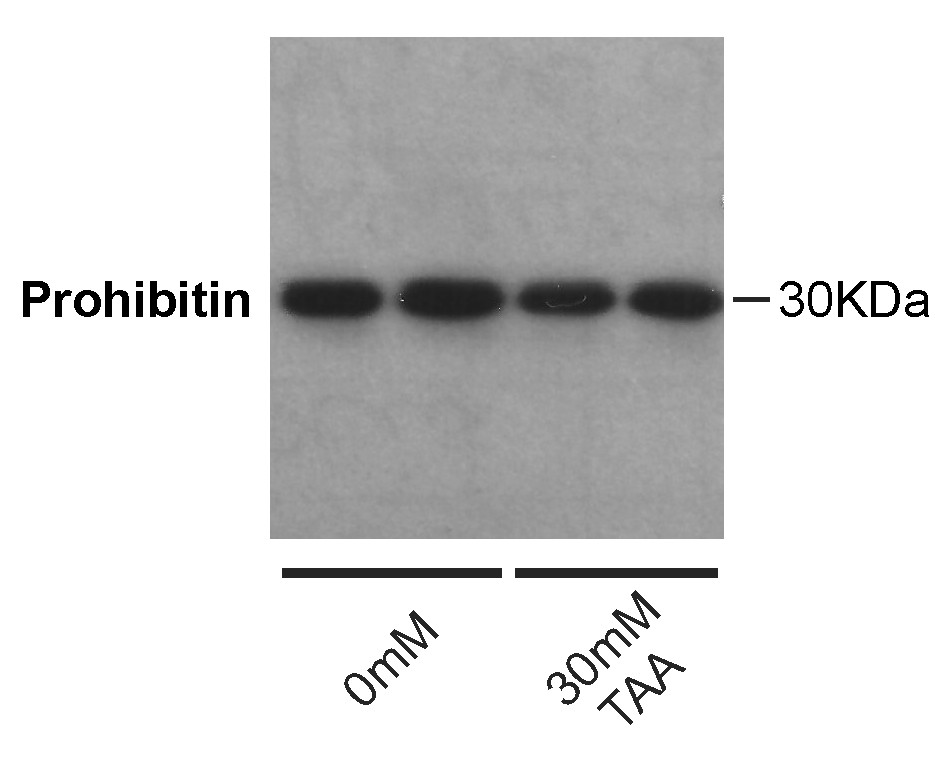

Supplement: Supplementary file 2 [file mmc2.zip › Raw data Fig.1B immunoblotting prohibitin.tif]
